# Multiple fractures due to hungry bone syndrome following parathyroidectomy: a clinical case report and review of literature

**DOI:** 10.1186/s40842-024-00183-8

**Published:** 2024-08-17

**Authors:** Farnaz Tavakoli, Fatemeh Yaghoubi, Davood Dalil, Mahdi Rezaei

**Affiliations:** 1 Department of Internal Medicine, Shariati Hospital, School of Medicine, Tehran University of Medical Sciences, Tehran, Iran; 2 Student Research Committee, Faculty of Medicine, Shahed University, Tehran, Iran

**Keywords:** Tertiary hyperparathyroidism, Parathyroid adenoma, Parathyroidectomy, Hungry bone syndrome, Hemodialysis, ESRD

## Abstract

**Background:**

Hungry bone syndrome (HBS) is defined as prolonged hypocalcemia caused by a sudden decrease in parathyroid hormone (PTH) levels after parathyroidectomy (PTX). Multiple fractures after PTX due to HBS in an end-stage renal disease (ESRD) patient on chronic hemodialysis (HD) are challenging and rare medical conditions presented in this study.

**Case Presentation:**

A 42-year-old ESRD patient on HD 3 times a week presented to Shariati Hospital, Tehran, Iran, complaining of worsening bone pain and loss of appetite. Laboratory data revealed an intact parathyroid hormone (iPTH) concentration of 2500 pg/mL, an alkaline phosphatase (Alp) level of 4340 IU/L, a phosphorus (P) level of 9 mg/dL, and a calcium (Ca) concentration of 7.2 mg/dL. Sestamibi scintigraphy revealed parathyroid adenoma. The findings suggested tertiary hyperparathyroidism (HPT-III), and the patient was scheduled for total PTX. Approximately one month after surgery, the patient was referred due to convulsions, leg mobility problems, and worsening bone pain. There was bilateral femoral ecchymosis. The Ca concentration was 5.8 mg/dL, and radiological evaluations revealed multiple skeletal fractures. HBS after PTX was suggested for this patient. After several days of hospitalization, he suffered subcutaneous emphysema followed by rib fractures and passed away.

**Conclusions:**

Multiple fractures after PTX due to HBS following HPT-III in ESRD patients are rare and demanding, highlighting the necessity of timely diagnosis and management of patients with HPT-III. Severe hypocalcemia following PTX can cause skeletal disorders. However, the surgical treatment of parathyroid adenomas may be more important than the risk of complications associated with bone health.

## Background

Tertiary hyperparathyroidism (HPT-III) is characterized by the autonomous functioning of parathyroid glands due to long-lasting stimulation following prolonged secondary hyperparathyroidism in patients with end-stage renal disease (ESRD) on dialysis or even after a successful renal transplant [1]. Parathyroidectomy (PTX) has been shown to be more efficient than medical therapy with cinacalcet for the treatment of HPT-III. However, only 7% of patients with HPT-III undergo PTX [2].

Hungry bone syndrome (HBS) is commonly defined as severe and prolonged postoperative hypocalcemia caused by a sudden decrease in parathyroid hormone (PTH) levels after PTX. Very low levels of serum PTH can negatively affect bone mineral density, causing adynamic bone disease or fractures [3]. It was previously reported that the risk of fracture among dialysis patients after PTX was lower than that in patients matched for age, sex, and race [4]. However, the risk rates and types of fractures after PTX are debatable. Here, we present a rare case of multiple fractures after PTX following HPT-III in a patient on chronic hemodialysis (HD).

## Case presentation

**Fig. 1:**
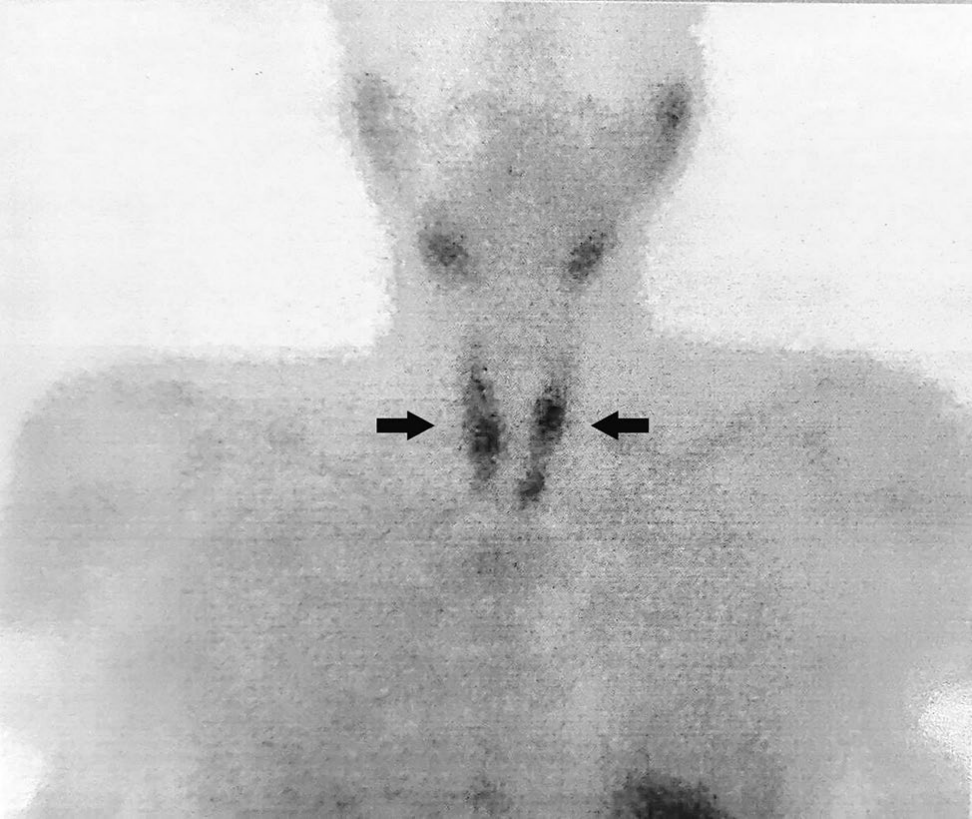
Sestamibi scintigraphy demonstrates increased uptake in all parathyroid glands, suggesting multiple parathyroid adenomas

A 42-year-old man who presented to Shariati Hospital, Tehran, Iran, complained of worsening bone pain and loss of appetite. His medical history included kidney stones, hypothyroidism, and ESRD of unknown cause, and he had undergone HD treatment three times a week for the last year. In addition, he was not an active smoker. Considering his past medical records, complementary diagnostic tests were requested. We evaluated the patient for chronic kidney disease mineral bone disease (CKD-MBD), which revealed an intact PTH (iPTH) level of 2500 pg/mL, an alkaline phosphatase (Alp) level of 4340 IU/L, a phosphorus (P) level of 9 mg/dL, and a calcium (Ca) concentration of 7.2 mg/dL. Sestamibi scintigraphy was then performed, which revealed parathyroid adenomas (Fig. 1). Based on these findings, a diagnosis of tertiary hyperparathyroidism was proposed. Consequently, the patient was scheduled to undergo total PTX. The postoperative iPTH levels were 345 pg/mL and 253 pg/mL at 10 min and 20 min after surgery, respectively. The calcium level decreased to 6.4 mg/dL.

To prevent HBS, pre, peri-, and postoperative calcium gluconate was administered intravenously, followed by the addition of oral Ca supplements to the treatment regimen. The patient was discharged 10 days after surgery with a Ca concentration of 7.5 mg/dL and a P concentration of 1.9 mg/dL. We recommended continuing treatment with calcitriol, calcium forte, and calcium-D and measuring calcium twice a week before dialysis.

Approximately three weeks later, the patient was referred to this center because of convulsions during dialysis, severe mobility problems in his legs, and worsening bone pain. He admitted that he had not taken the prescribed medication since being discharged from the hospital. Upon examination, bilateral femoral ecchymosis was observed. The initial blood test revealed a white blood cell (WBC) count of 17,300/mm3, a hemoglobin (Hb) concentration of 8.1 g/dL, an erythrocyte sedimentation rate (ESR) of 103 mm/hr, a blood urea nitrogen (BUN) level of 54 mg/dL, a creatinine (Cr) level of 8.5 mg/dL, a uric acid concentration of 6.6 mg/dL, a P concentration of 2.6 mg/dL, a Ca concentration of 5.8 mg/dL, a lactate dehydrogenase (LDH) level of 847 IU/L, a creatine phosphokinase (CPK) level of 5600 IU/L, an Alp level of 1668 IU/L, a 1,25-hydroxy vitamin D (vitamin D) concentration of 13 ng/mL, and an iPTH concentration of 9 pg/mL.

**Fig. 2:**
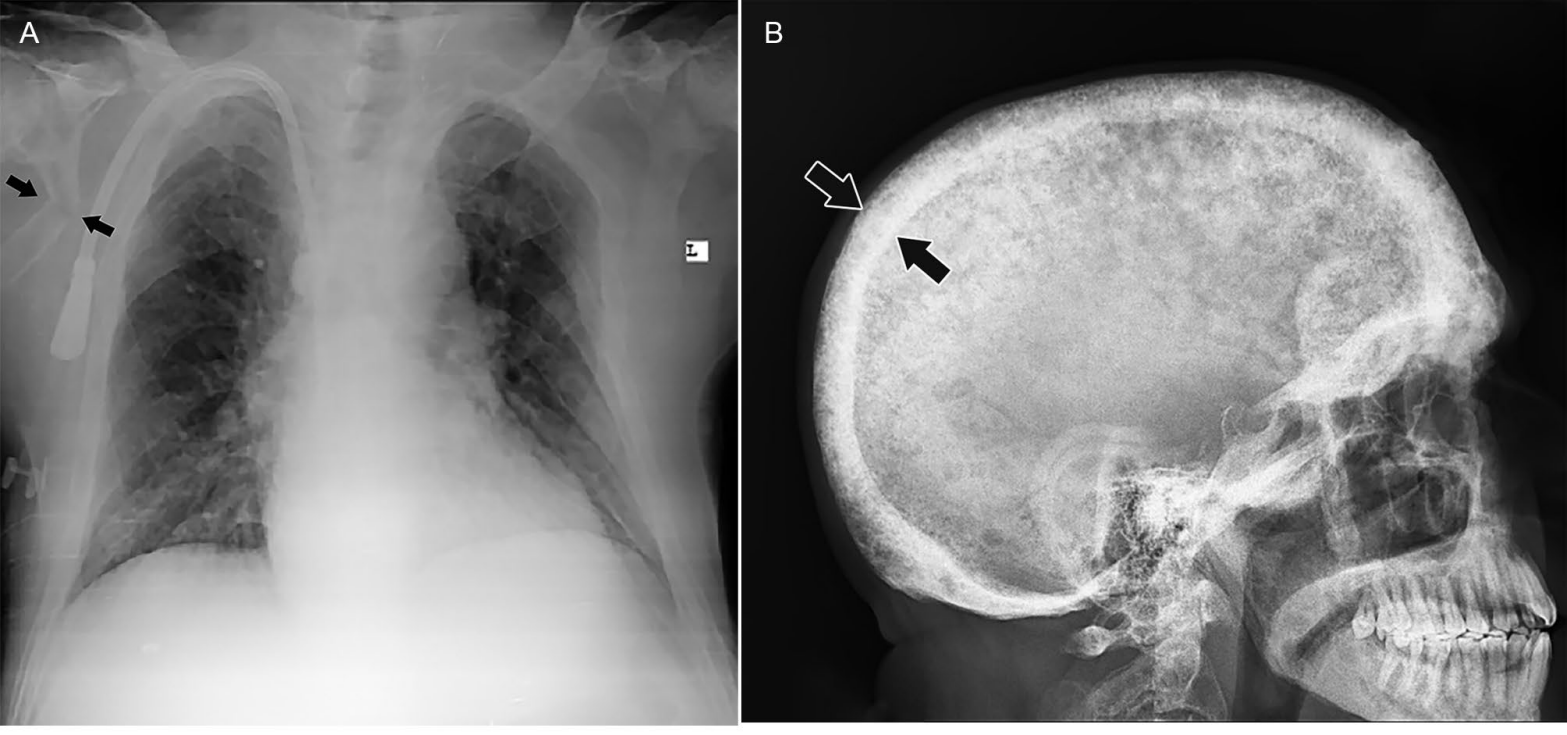
**A**: Fracture in the patient’s scapula; **B**: Diffuse thickening of the skull along with numerous lytic bone lesions in the calvarium

**Fig. 3:**
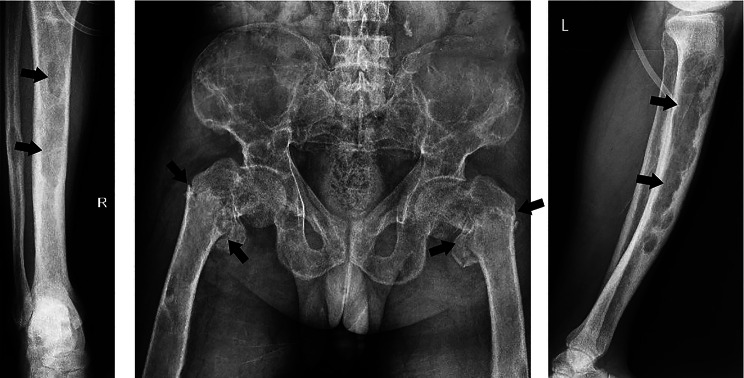
Pelvic and lower leg X-ray images; bilateral fractures in the intertrochanteric region of femur and diffused lytic lesions with mild to moderate expansion mainly in medullary bone

He was initially prescribed high doses of intravenous Ca followed by high doses of oral Ca. On radiological evaluation, a spiral brain computed tomography (CT) scan revealed diffuse thickening of the skull along with numerous lytic bone lesions in the calvarium. Spiral neck CT revealed diffuse punctate lytic lesions in the cervical spine. There were also diffuse lytic bony lesions and fractures in the patient’s scapula and thoracic spine, as reported on chest X-ray imaging (Fig. 2). Moreover, pelvic bone X-ray images revealed bilateral fractures in the intertrochanteric region of the femur and diffuse lytic lesions with mild to moderate expansion, mainly in the medullary bone (Fig. 3). After several days of hospitalization due to multiple fractures, he developed subcutaneous emphysema followed by rib fractures and died.

## Discussion

ESRD individuals struggle with various clinical problems throughout their lives [5, 6]. Chronic kidney disease (CKD) patients are at risk of secondary hyperparathyroidism (HPT-II) due to high PTH levels and various metabolic disturbances, such as calcitriol deficiency, hypocalcemia, and hyperphosphatemia [7, 8]. Parathyroid gland cells are directly stimulated by hyperphosphatemia, which causes nodular hyperplasia and increased PTH release, leading to persistent hypocalcemia [9]. In addition, HPT-II occasionally develops into a hypercalcemic state and excessive PTH secretion, similar to the autonomous type of hyperparathyroidism known as HPT-III, in ESRD patients undergoing chronic HD and/or kidney transplant recipients [10]. HPT-III occurs when the chief cells of the parathyroid glands secrete excess PTH to balance their function and respond to chronic hypocalcemia.

CKD-MBD assists physicians in the evaluation of CKD patients, those on chronic dialysis, and those who have had kidney transplants. Phosphate, Ca, and PTH levels may be related to mortality in CKD patients, as shown in CKD-MBD [11]. Furthermore, sestamibi scintigraphy has equivalent sensitivity to ultrasonography for detecting parathyroid adenomas, which is increased by concurrent tomographic imaging [12]. In this case, considering long-term ESRD and chronic HD, we evaluated the patient for CKD-MBD, which revealed highly elevated levels of iPTH, Alp, and P and decreased Ca levels. According to these findings, a sestamibi scan was performed, which indicated a parathyroid adenoma.

During the waiting period before transplantation, PTX is preferred for patients with HPT-III and hypercalcemia. Surgery is required because it normalizes the serum Ca concentration by reducing the parathyroid bulk and cell count [9]. Although there are currently no approved medicinal treatments prior to PTX, taking bisphosphonates and increasing vitamin D levels may help prevent complications, such as HBS. HBS refers to abrupt and prolonged hypocalcemia (lasting longer than four days postoperatively), usually followed by hypophosphatemia and hypomagnesemia after PTX. PTH levels drop immediately after removing one adenoma, and long-term suppression of the other glands ceases bone resorption. Continuous bone growth causes an increase in skeletal Ca consumption and the resulting hypocalcemia [3, 13].

Despite the lack of data, it is estimated that up to 13% of PTX patients experience HBS. Risk factors for developing HBS include younger age at the time of surgery, high levels of PTH and Alp, low levels of albumin and magnesium, and skeletal abnormalities, such as subperiosteal erosions, lytic lesions, and brown tumors [14]. Notably, our patient had several risk factors, including remarkably elevated PTH and Alp levels, along with multiple skeletal fractures.

Treatment for HBS aims to replenish the depleted skeletal Ca stores. For the treatment of severe hypocalcemia, 6–12 g/day Ca supplementation is frequently required [3, 14, 15]. PTH levels have been independently associated with a greater risk of fracture incidence [16, 17], with iPTH levels exceeding 900 pg/ml. For cases at high risk of HBS and severe osteoporosis, monitoring is advised to determine when to start Ca and calcitriol supplementation [18]. Another study stated that there is no link between vitamin D deficiency, HBS, or metabolic bone disease; but high preoperative iPTH increases HBS risk; and postoperative Ca and vitamin D supplementation recommended as cost-effective approach to restore normal bone metabolism [19]. Dalmazi et al. reported a case of parathyroid apoplexy caused by cinacalcet treatment in a patient with primary hyperparathyroidism and they call for additional research on managing primary hyperparathyroidism patients undergoing cinacalcet treatment [20]. Our patient’s PTH level was 2500 pg/ml, and a high dose of intravenous and oral Ca was prescribed for treatment.

Adults with HPT-III comprise 4.5% of those who experience bone abnormalities such as fractures and brown tumors [21]. In people with aberrant bone structures caused by HBS, pathological fractures can occur with minor damage; as a result, a proper diagnosis is crucial to prevent fractures [22]. The scapula, spine, and intertrochanteric regions of the femur were fractured in our patient.

There is still a high rate of morbidity and mortality associated with rib fractures in individuals with thoracic trauma because of underlying lung and heart injuries that cause additional pulmonary issues. Among patients with multiple fractures, rib fractures are associated with a worse prognosis because they are more likely to cause respiratory compromise and coexisting injuries [23]. After several days of hospitalization due to multiple fractures, our patient developed subcutaneous emphysema followed by rib fractures and died.

Moreover, we searched the PubMed/Medline database for English-published literature of reported cases developing HBS after PTX during the last decade, resulting in 24 articles, shown in Table 1 [24–47]. These studies cover a total of 27 patients from the years 2014 to 2024. The distribution of male and female patients was nearly equal, with males comprising 14 out of the 27 total cases documented. The age range of patients is quite broad, from 12 to 74 years old, indicating the diverse age groups are at risk of HBS after PTX. Of all the patients, 19 individuals diagnosed with primary hyperparathyroidism due to parathyroid adenoma or parathyroid carcinoma. These patients regularly presented with bone pains, fatigue and weakness. Similar to our case, five cases were on chronic HD. There is no major difference in the choice of treatment method and the type of drug given; And it is a combination of Ca and vitamin D supplements. Ca-citrate was reported to be more effective in improving hypocalcemia in patients who used proton pump inhibitors and Ca supplementation together [30].

**Table 1:** Cases of developing HBS after PTX

Author (Year)	Sex/Age	Presentation	Comorbidities	Diagnosis	Bone Radiography	Complication	Treatment of HBS
Varma et al. (2014) [24]	F/36	HyperCa & PTH crisis	N/A	P-HPT due to PC	Lytic lesions in the right femoral neck, right Sup. and Inf. pubic bones and left iliac wing	Osteitis fibrosa cystica	IV and oral Ca
Neagoe et al. (2014) [25]	M/57	Osteoarticular pains, muscular weakness, nausea & abdominal pains	Renal stones, Osteoporosis, HLP, IHD, Prostate adenoma	P-HPT due to PA	N/A	None	IV Ca-gluconate, Oral Alfacalcidol
	F/60	Fatigue, muscular weakness, nausea, vomiting & dehydration	Hypothyroidism, Peptic disease	P-HPT due to PA	N/A	None	Oral Ca
Altun et al. (2015) [25]	F/20	HyperP, HypoCa & PTH crisis	ESRD on HD, Anuria	HPT-II without PA	N/A	Prolonged HypoP	IV Ca, Oral CaCO_3_ & calcitriol
	F/32	HypoCa & PTH crisis	CTIN secondary to NBSD, HD, Anuria				
	M/35	PTH crisis	AA amyloidosis secondary to FMF, HD, Anuria				
Ebina et al. (2015) [27]	M/16	Hip & right forearm pain	None	P-HPT due to PA	Lytic fractured lesion in both femoral neck and radial shaft	Multiple brown tumors & secondary osteoporosis	IV Ca & Mg, Oral Ca & calcitriol
Saif et al. (2015) [28]	F/15	Recurrent vomiting & abdominal pains	None	P-HPT due to PA	Osteopenia and lytic areas in the left iliac bone, the femoral neck bilaterally, and the right pubic bone	HyperCa-induced pancreatitis	IV & oral Ca, Oral Alfacalcidol
Rutledge et al. (2016) [29]	F/21	Enlarging right-sided neck mass	None	P-HPT due to APA	No osseous abnormalities	None	IV Ca, Oral Alfacalcidol, Oral P & Mg
Afshan et al. (2017) [30]	M/32	Perioral numbness, tingling, diffuse paresthesia & weakness	ESRD on PD, HTN, GERD	N/A	N/A	Severe & recurring HypoCa	Changing oral CaCO_3_ to Ca-citrate with oral clcitriol
Hendarto et al. (2017) [31]	F/18	Generalized bone pain	None	P-HPT due to PA	Severe scoliosis, multiple old fractures, bone erosions and resorption of the phalanges	None	Parenteral & oral Ca
Tai et al. (2018) [32]	F/56	Frequent urination, generalized weakness & pain	HTN, HLP, DM	P-HPT due to PA	Triangular fibrocartilage complex chondrocalcinosis	Pseudogout	IV Ca & Mg, Colchicine, Naproxen
Florakis et al. (2019) [33]	M/44	Chronic arthralgia & myalgia	None	P-HPT due to APA	Diffuse osteopenia of hip-pelvis and lumbar spine	HPT-II due to vit D deficiency	Alfacalcidol, Ca, vit D and Mg
Tai et al. (2019) [34]	M/74	HyperP, HyperCa & PTH crisis	ESRD on HD, HTN	HPT-III due to PG & ectopic PG	N/A	None	IV Ca-chloride, Oral Ca-acetate and vit D
Ahmed et al. (2020) [35]	F/35	HypoCa & PTH crisis	SLE, on HD	HPT-II	N/A	None	IV Ca & vit D, Oral Ca & teriparatide
Buisset et al. (2020) [36]	F/20	HyperCa & PTH crisis	None	P-HPT due to PC	Diffuse bone demineralization	Osteitis fibrosa cystica & multiple brown tumors	Oral Ca & vit D
Lenherr-Taube et al. (2020) [37]	F/13	HyperCa & PTH crisis	Bilateral genu varum, Hip impingement	P-HPT due to PA	Lytic lesions throughout the skeleton and widening of the bilateral sacroiliac joints	None	IV Ca, Oral CaCO_3_ & calcitriol
Raj et al. (2020) [38]	F/66	HypoCa & PTH crisis & fatigue	Renal stones	P-HPT due to PA	N/A	HPT-II due to CKD	IV Ca-gluconate, Oral Ca, calcitriol & Ergocalciferol
Alvarez et al. (2022) [39]	F/55	Mechanical pain in the right shoulder & loss of strength	Osteoarthritis, HTN	P-HPT due to PA	Lytic lesions on the proximal humerus, sternum, acromion, sixth anterior left costal arch, iliac bones, left femoral head, and fracture of the Sup. vertebral disc	Acute and chronic pulmonary embolisms	IV Ca-gluconate, Oral calcitriol & Ca
Landeta et al. (2022) [40]	M/56	HyperP, HypoCa & PTH crisis	HTN, BPH	P-HPT due to PA	N/A	None	IV Ca-gluconate, Oral CaCO_3_
Vitale et al. (2022) [41]	M/12	Nausea, HyperCa & PTH crisis	Obesity, Autism spectrum disorder	P-HPT due to PA	N/A	None	Oral Ca & calcitriol
Zelano et al. (2022) [42]	M/56	HypoCa, fatigue, abdominal pain, vomiting, dehydration & weight loss	None	P-HPT due to PC	Calcification in the cartilage around the joints, associated with synovitis in hands, wrists, elbows, knees, and ankles	Articular pain & pancreatic exocrine insufficiency	IV Ca, Oral calcitriol
Martínez‑Loya et al. (2023) [43]	M/34	Muscle weakness	Bilateral hip fractures	P-HPT due to PA	N/A	None	IV Ca, Oral calcitriol & CaCO_3_
Nievera et al. (2023) [44]	M/21	Severe pain of both thighs & his legs give way	None	P-HPT due to PA	Displaced fractures of both upper femoral shafts	None	IV Ca-gluconate, Oral Ca
Shah et al. (2023) [45]	M/64	Inability to walk for nine days & bilateral knee pain radiating to shins	HTN, DM	P-HPT due to PA	N/A	None	Oral calcitriol & CaCO_3_
Liu et al. (2024) [46]	M/57	Multiple pathological fractures, muscle atrophy & bone pain	None	P-HPT due to PC	Femoral neck fracture and muscle atrophy around the left hip, bilateral humeral shaft fractures	Numbness in perioral sites and fingertips	IV Ca-gluconate, Oral Vit D
Papanikos et al. (2024) [47]	M/67	HyperCa, nausea, drowsiness, & weakness	HTN, HLP, Hyperuricemia	P-HPT due to PA	N/A	None	IV Ca-gluconate, Oral Alfacalcidol

**HBS**, Hungry bone syndrome; **PTX**, Parathyroidectomy; **HyperCa**, Hypercalcemia; **PTH**, Parathyroid hormone; **P-HPT**, Primary hyperparathyroidism; **PC**, Parathyroid carcinoma; **Sup**, Superior; **Inf**, Inferior; **IV**, Intravenous; **Ca**, Calcium; **HLP**, Hyperlipidemia; **IHD**, Ischemic heart disease; **PA**, Parathyroid adenoma; **HyperP**, Hyperphosphatemia; **HypoCa**, Hypocalcemia; **HPT-II**, secondary hyperparathyroidism; **ESRD**, End stage renal disease; **HD**, Hemodialysis; **CTIN**, Chronic tubulointerstitial nephritis; **NBSD**, Neurogenic bladder sphincter dysfunction; **AA**, Amyloid A; **FMF**, Familial Mediterranean fever; **HypoP**, Hypophosphatemia; **CaCO**_**3**_, Calcium carbonate; **Mg**, Magnesium; **APA**, Atypical parathyroid adenoma; P, Phosphorus; **PD**, Peritoneal dialysis; **HTN**, Hypertension; **GERD**, Gastroesophageal reflux disease; **SLE**, Systemic lupus erythematosus; **DM**, Diabetes mellitus; **PG**, Parathyroid gland; **CKD**, Chronic kidney disease; **BPH**, Benign prostate hyperplasia.

## Conclusion

To our knowledge, the present case is a rare case of multiple fractures after PTX due to HBS following HPT-III and multiple parathyroid adenomas in a patient with ESRD on chronic HD. This case may provide insight into the diagnosis and management of HPT-III. Patients with HPT-III may experience bone pain or fractures due to osteopenia or osteoporosis, respectively. Moreover, severe hypocalcemia following PTX can cause skeletal disorders. However, the surgical treatment of parathyroid adenomas may be more important than the risk of complications associated with bone health.

## Data Availability

Not applicable.
